# Quality Considerations When Using Tissue Samples for Biomarker Studies in Cancer Research

**DOI:** 10.1177/11772719211009513

**Published:** 2021-04-20

**Authors:** Valerie Speirs

**Affiliations:** Institute of Medical Sciences, School of Medicine, Medical Sciences and Nutrition, University of Aberdeen, Aberdeen, Scotland, UK

**Keywords:** Biomarker, biobank, cancer, outcome, data, informatics

## Abstract

Tissue obtained from biobanks is frequently employed in biomarker studies. Biomarkers define objective, measurable characteristics of biological and biomedical procedures and have been used as indicators of clinical outcome. This article outlines some of the steps scientists should consider when embarking on biomarker research in cancer research using samples from biobanks and the importance and challenges of linking clinical data to biological samples.

## Introduction

At the start of my scientific career, nearly 3 decades ago, tissue provision for research was seen as the preserve of surgeons with tissues obtained directly from the operating theatres. Requirements for ethical approval were not as rigorous as they are nowadays. Pathology was bypassed completely; you knew approximately when a patient was going to be in theatre and someone from the lab team, usually the most junior member, would be waiting patiently outside to obtain a tissue sample. If you were lucky, a theatre nurse would emerge sooner or later with said tissue sample in a pre-labelled tube containing transport medium that the lab had previously provided. More often than not however, the tissue would be provided dry in a miscellaneous tube rustled up by the operating theatre team, labelled with the tissue type (if you were lucky), and often bore the bloody thumbprint of the surgeon who had performed the operation on the side of the tube. Tissue would be transported back to the lab where snap frozen aliquots would be prepared, to be stored in a −80 freezer, which held an assortment of frequently accessed reagents as well as tissue samples. Temperature control and monitoring was unheard of, leaving stored tissues vulnerable to temperature fluctuations as various people accessed the freezer throughout the day. In these days tissue collections were frequently driven by the research interests of individuals and it was not uncommon for these to be viewed as personal fiefdoms. On the rare occasion that tissue samples were, often grudgingly, released from the grasp of the ‘owner’, the grateful recipient was often left thinking; How was the sample collected? Who collected it? How was the tissue sample stored? How was it catalogued? Nowadays, the situation is very different. Scientists have realised that tissue collected, processed and stored on an ad hoc basis is now insufficient for biomarker studies in cancer research, resulting in the evolution of ‘biobank fiefdoms’ into the much more professional biobanks we are familiar with today. Tissues are not collected without appropriate ethics being in place and with the written informed consent of the donor. Pathologists are now, quite rightly, front and centre in cancer biobanking activities. Over the years, samples collected by biobanks have expanded from just tissue samples alone, to include tissue derivatives such as DNA, RNA and protein, as well as whole blood serum and plasma, plus other biofluids. Adding corresponding clinical data to match these samples means biobanking is now a complex ecosystem. With research reproducibility now high on the scientific agenda,^1,2^ twinned with a substantial increase over the last 1 to 2 decades in the need for good quality, well-annotated tissue samples, from both academia and industry, has initiated this agenda for change.

## Biomarker Studies in Cancer

Tissue obtained from biobanks is frequently employed in biomarker studies. The noun ‘biomarker’ is a relatively recent addition to the biomedical dictionary and is a portmanteau of ‘biological marker’. Biomarkers define objective, measurable characteristics of biological and biomedical procedures and have been used as indicators of clinical outcome. Consequently, there has been much interest in identifying new biomarkers with the hope that these may make it to the clinical arena at some point in the future. While the cornerstone for biomarker studies in cancer has been immunohistochemical detection in formalin-fixed, paraffin-embedded (FFPE) tissues, there are now many types of cancer biomarkers, either in use in clinical practice currently, or with the potential to be used in future. These are summarised in Table 1. Perhaps the most well-known and well-validated biomarker employed routinely on FFPE tissue sections is the oestrogen receptor (ER), which is both a predictive and a prognostic biomarker in breast cancer; its presence in the nuclei of FFPE breast tumour sections can help clinicians predict clinical outcome as well as the likelihood of patient response to adjuvant endocrine therapy. However, before being of clinical value, reproducibility is a critical quality that all biomarkers must meet; as a tried and tested biomarker over many years, ER meets these criteria easily.

**Table 1.** Examples of current and emerging cancer biomarkers.

Biomarker type	Assay for measurement	Clinical example (cancer type)^a^
Protein	IHC	Oestrogen receptor (breast cancer)
DNA	FISH, CISH	*BCR*/*ABL* (chronic myeloid leukaemia)
RNA	Gene expression	*KRAS* (colon cancer)
Blood	ELISA	Prostate-specific antigen (prostate cancer)
Plasma	ELISA	CEA (colon cancer)
ctDNA^b^	Droplet digital PCR Sequencing	Potential for monitoring disease status of multiple cancer types
Blood	ELISA	Prostate-specific antigen (prostate cancer)
Plasma	ELISA	CEA (colon cancer)
miRNA^b^	PCR, gene array, RNA-seq	Potential cancer biomarker
lncRNA^b^	PCR	Potential cancer biomarker
Exosomes^b^	PCR, following enrichment from blood	Potential cancer biomarker

Abbreviations: CEA, carcinoembryonic antigen; CISH, chromogenic in situ hybridisation; ELISA, Enzyme-linked immunosorbent assay; FISH, fluorescent in situ hybridisation; IHC, immunohistochemistry; lncRNA, long non-coding RNA; miRNA, microRNA; PCR, polymerase chain reaction.

a Non-exhaustive; single representative examples are provided.

b Emerging biomarkers; not used in clinical practice currently.

At the other end of the spectrum, the most recent type of biomarker which is showing the greatest potential to enter the clinical arena is circulating tumour DNA (ctDNA). This is often referred to as a liquid biopsy as ctDNA is detectable in blood plasma where it has been shown not only to reflect the mutational signatures of the primary tumour, which may guide treatment,^3,4^ but also as an non-invasive biomarker, for early cancer detection or to monitor tumour progression following treatment.^5-7^ In contrast to traditional tissue-based biopsies, which are generally not are not amenable to repeated sampling, and only provide a snapshot of a tumours composition, the liquid biopsy has the advantage of being taken at multiple points in the patient management pathway, and can be used a as a surrogate to allow changes in the genetic composition of a tumour to be determined more easily.^8^

## Do We Need an Expiry Date for Biobank Samples Used in Translational Cancer Research?

We are all familiar with ‘best before’, ‘use by’ and ‘sell by’ dates on perishable and even some frozen foodstuffs yet there is little directive on whether these same measures should be applied to tissue samples in biobanks. As demonstrated in Table 2, there are multiple examples of biobank standards, covering the tissue, collection, processing and storage yet literature suggests that the range and type of quality control (QC) procedures employed by biobanks is often limited.^9^ Commonly, QC/quality assurance measures are limited to examination of tissue by a pathologist to confirm histopathological diagnosis, the presence and percentage of tumour, necrosis and immune infiltration. While this is certainly helpful, quantitative metrics regarding the impact of ex vivo ischaemic and storage times on tissue morphology, especially when frozen, DNA and RNA integrity (RIN) and expression/integrity of housekeeping proteins, would be useful in guiding scientists when selecting tissue samples for specific research purposes. While these may be well documented by biobanks, they are often not easy to find by end users. Instead, informal metrics of tissue quality including testimony from previous users, acknowledgement of the biobank which has provided tissue samples in a published research paper or presentation tend to be used. However, unless these metrics are displayed on a biobank website, these are often hard to track down.

**Table 2.** Resources for best practice guidelines for biobanks.

Organisation	Location	Resource	Reference
Australian biobank accreditation scheme	Australia	Biobank certification programme	Ling et al^10^ and Carpenter et al^11^
Biobanking and biomolecular resources (BBMRI)	Europe	Research infrastructure for biobanking	Litton^12^
College of American pathologists (CAP)	US	Biorepository Accreditation Program	McCall et al^13^ and Barnes et al^14^
National cancer research institute cellular molecular pathology (NCRI CMPath)	UK	Biobanking Sample Quality Improvement Tool which can be downloaded and used freely	Speirs et al^15^
Canadian tissue repository network (CTRNet)	Canada	Biobanking process and data standards	Barnes et al^14^ and Hartman et al^16,17^
International society for biological and environmental repositories (ISBER)	International	Suite of tools (Self-Assessment Tool is restricted to members only)	Campbell et al^18^ and ISBER^19^
Biospecimen pre-analytical variables (BPV)	US	Suite of Standard Operating Procedures to assist biobanks which can be downloaded freely	Hartman et al^16^ and Carithers et al^20^

Some studies have addressed the impact of long-term storage of tissues in biobanks specifically. A comparative study of 12 phosphoprotein epitopes, evaluated on tissue microarrays of FFPE breast tumours, as a function of time to fixation, showed change in expression of most epitopes with increasing time to formalin fixation.^21^ A study evaluating the robustness of EndoPredict, a quantitative PCR assay that uses RNA extracted from FFPE tissue to predict the likelihood of distant recurrence in patients with ER + HER2- breast cancer, showed no impact of pre-analytical tissue handling on gene expression in this assay.^22^ Of nearly 400 fresh frozen tissue samples obtained from different tumour-types obtained from the Samsung Medical Center Biobank showed that cold ischaemic times of up to 1 hour and storage times of up to 6 years did not impact adversely on RNA integrity.^23^ An evaluation of ER expression in FFPE breast cancer cases dating from the 1940s to 2000s, using standard immunohistochemical methods, showed that ER could be detected across the decades, with no appreciable change in quality of signal.^24^ Similarly storage of gastric cancer samples at −80°C for 12 years did not adversely affect RNA integrity or tissue morphology, although RIN was influenced by cellular composition.^25^ When RIN numbers were high, RNA quality was not compromised in pancreatic cancer samples that had been snap frozen at various timepoints of up to 1 hour post-surgical resection,^26^ however as ischaemic time increased, RIN number decreased.^26^ In the US, the Biospecimen Preanalytical Variables Program (BPV),^20^ was instigated by the National Cancer Institute. Recognising that FFPE samples are used frequently in biomarker studies the BPV evaluated the impact of tissue processing on the quantity and quality of nucleic acids isolated from FFPE colon, kidney and ovarian tumours and compared this to the same tissue which had been snap-frozen. Results showed that fixations delays of up to 12 hours and time in fixative of 72 hours adversely affected DNA and RNA quality.^20^

Most of the studies outlined above have studied samples collected and stored by same biobank. However, scientists often need to source samples from multiple biobanks to obtain sufficient numbers to make their results statistically robust. This is especially true when studying a rare cancer type/subtype. Furthermore, of the biomarkers that do show promise in initial, discovery science phases, journals demand that these be validated in independent cohorts before there is any chance of them being adopted in the clinic. As validation samples are frequently sourced from other biobanks, it is not hard to imagine how even small variations in sample processing and storage between discovery and validation cohorts may contribute to irreproducible data. Indeed it is clearly recognised that such variables are key issues associated with a lack of reproducibility of results.^27^ The Breast Cancer Now Tissue Bank (BCNTB) was established in the UK a decade ago and operates as a hub and spoke model, with tissue collected according to standard SOPs at various constituent centres.^28^ During this time, it has completed 2 cycles of QC work designed to test the quality of stored samples collected at different centres, including impact of storage time, ischaemic interval, RIN, tissue integrity and immunohistochemistry and a manuscript is in preparation.

Findmyassay.com,^29^ developed by the Integrated BioBank of Luxembourg^30^ is a free online resource which offers a different approach for scientists to help identify the suitability of previously collected tissues for different downstream analyses. Its algorithm suggests a range of tests that can be performed on tissues, designed as surrogates to assess analytical variables such as fixation time and conditions, cold ischaemia, percentage tumour and fitness for purpose of sample for immunohistochemistry. However, this requires additional tissue to perform these analyses and may not be always be practical, especially when tissue is limiting.

## Quality Guidelines and Standards

It is well-recognised that biomarker studies in cancer can be adversely affected by confounding variables such as the time a biopsy was taken, for example, pre- or post-surgical resection.^31^ and the inflammatory status of the tissue.^32^ As a result, appeals for have been made for standardisation to ensure biomarkers can be reliably and consistently measured.^33^ This complements desires to improve quality standards in biomedical research. Consequently, and mindful of the need for transparent reporting mechanisms to drive scientific reproducibility, best practice guidelines for various scientific procedures have been established over the years. Many fall under the umbrella of EQUATOR (Enhancing the QUAlity and Transparency Of health Research Network), an international network designed to improve the reliability and consistency of published literature in the health research sector.^34^

One of the first sets of guidelines came about in response to the burgeoning use of microarray analysis which was generating large volumes of gene expression data on a genomic scale at the start of this millennium, and resulted in the introduction of MIAME (Minimum Information About a Microarray Experiment).^35^ These described the minimum information that was needed to ensure that published microarray data could be easily understood and that results derived from its analysis could be independently confirmed. Subsequently, the advent of next-generation sequencing resulted in the introduction of MINSEQE^36^ (Minimum Information about a high-throughput nucleotide SEQuencing Experiment), covering sequencing data. Compliance with both sets of guidelines is recommended when depositing data describing microarray or sequencing studies to The National Center for Biotechnology Information (NCBI) Gene Expression Omnibus (GEO) and many journals mandate this before accepting a manuscript for publication. In animal research, the ARRIVE (Animal Research: Reporting of In Vivo Experiments) guidelines were introduced, comprising a 20-point checklist of essential information to include in publications which use animals in research, as a means of improving the design, implementation and reporting of such studies^37^ and have recently been refreshed.^38^ With all the above guidelines being widely endorsed by journals, they are frequently reported in original research articles, serving as benchmarks for study quality.

Guidelines exist which are pertinent to biobanking. These include are REMARK (REporting recommendations for tumour MARKer prognostic studies),^39^ SPREC (Standard PREanalytical Code)^40^ and BRISQ (Biospecimen Reporting for Improved Study Quality).^41^ REMARK was introduced as a means of improving reporting of biomarker studies through recognition that despite several hundreds of reports on potential prognostic tumour biomarkers, vanishingly few made it into clinical practice.^39^ SPREC (Standard PREanalytical Code) guidelines were introduced specifically for biobanking to identify and regulate the main pre-analytical factors which might impact on sample quality during the sample collection timeline.^40^ Following a half-day workshop ‘Development of Biospecimen Reporting Criteria for Publications’, held just over a decade ago, where experts discussed optimum ways to collect, process and store human biospecimens for cancer research, Moore et al^41^ outlined the lifespan of a human biospecimen, from patient diagnosis and surgical intervention; sample acquisition, handing and processing; through to tissue distribution and scientific analysis, concluding with how to deal with any unused samples.^41^ Recognising the important, and often varied, pre-analytical variables at each of these steps, this work expanded SPREC into BRISQ, aiming to provide further standardisation and consistency for tissue-based research.^41^ Knowing that not all pre-analytical variables may be recorded by biobanks however, BRISQ adopted a tiered approach. Tier 1 includes items recommended to report; the organ or tissue, how it was collected stabilised and preserved, Tier 2 includes items beneficial to report; time from specimen excision and acquisition to stabilisation while Tier 3 includes additional items which are recommended to report; storage vessels, environmental factors. Despite this good work, while many journals mandate that REMARK criteria are provided when submitting a biomarker study for publication, somewhat surprisingly, the same has not (yet) been implemented for BRISQ.^42^ The onus is journal editors to facilitate this to improve the standard of submissions and ensure reproducibility. Recently the UK National Cancer Research Institute (NCRI) Cellular Molecular Pathology (CMPath) initiative developed a free online sample quality improvement tool.^15^ This confidential tool provides scientists with a means of self-assessing their current practices surrounding tissue collection, also providing guidance on areas which could be improved to increase the quality of tissue samples being collected for use in biomarker research.

## Linking Clinical Data to Biological Samples

An essential criterion for any modern biobank is the ability to link research findings derived from tissue samples to robust clinical and pathological data. Simply providing tissue or other biosamples is no longer sufficient for translational research. This is essential in biomarker studies, in order that scientists can link the presence or absence of a biomarker with clinical outcome. Many biobanks now collect comprehensive information including patient, pathology and clinical data, all carefully mined from clinical databases. Examples of the types of essential and desirable information requested by researchers is summarised in Table 3. This typically includes pathological data collected and reported according to published national guidelines, treatment type (eg, hormone, chemotherapy, radiotherapy or biological therapy), duration of and response to therapy, dates of local, regional and distant recurrence, as well as the date and cause of death plus other comorbidities. However, the challenges surrounding data linkage from electronic health records and informatics for biobanks are well recognised,^43,44^ particularly as academic institutes, healthcare systems and biobank networks are frequently separate entities, which may use different informatics platforms which cannot be linked easily. While steps are now being taken by various biobanks to make this process more streamlined,^45-47^ manual intervention is often still required making this an inefficient and time consuming process.

**Table 3.** Recommended essential and desirable data to accompany clinical samples obtained from biobanks.

Data category	Essential	Desirable
Donor	Age	Comorbidities
	Diagnosis	Family history
	Gender	Previous cancer smoking history
		Weight
Pathology	Grade	Whole slide digital image
	Histology	
	Stage	
	Receptor status	
	Tumour type	
Molecular	Gene mutations	Sequencing data
	Gene amplifications	
Treatment	Surgery	Treatment start and end dates
	Chemotherapy	Toxicities relating to treatment
	Radiotherapy	
Outcome	Survival status	Metastatic site
	Recurrence	
	Date of death	

Some biobanks make it a condition of use that data derived from samples is returned to the biobank upon completion/publication of a study meaning in some instances, this additional information may be available, for example, previous biomarker data, gene expression profiles, etc derived from the same patient samples, which can add to the richness of the data which can be obtained from donated tissues, for example, The Susan G. Komen Tissue Bank operates this model.^48^ In addition, the digital pathology revolution has facilitated the provision of H&E stained FFPE and frozen tissue sections in digital format. These images are typically hosted centrally in a biobank database with online access provided to researchers as required. Researchers can apply various Open Source software to these digital, whole slide images, for example, RandomSpot^49^ to assess tumour stroma ratio,^50^ QuPath^51^ to quantify adipocytes.^52^ More recently sophisticated deep learning approaches have been applied to standard histology sections, which have demonstrated that routine biomarkers like ER and clinically actionable molecular alterations can be predicted from routine histology slides.^53-55^ These exciting advances add to the breadth of information that can now be uncovered from tissue and its derivatives.

## Conclusions

As patient-centred, personalised medicine approaches start to be implemented more widely, allowing clinicians to determine with precision which treatment strategies will work for which cancer and in which patients, demands for high quality, well-annotated biospecimens that can be provided with confidence for robust biomarker research will only increase. Furthermore, tissue requirements from cancer researchers are becoming more complex, often requiring, from the same patient, longitudinal samples, primary and metastatic samples, matched tumour and normal samples^56^ and, from personal experience, all, or combinations of these. The need to link lab results to patient electronic health records presents additional challenges, with demands for this becoming increasingly complex too. Biobanks must continue to evolve to meet these demands, becoming more strategic in the range and type of biosamples they collect, with continued horizon scanning to remain agile to future research needs.

Finally, biomarker studies would not be possible without the ability to use samples from patients who selflessly and generously donate these with the sole intention that these are used to improve existing or develop new treatments for future cancer patients. We have a moral obligation to ensure these samples remain fit for purpose such that patient wishes are fulfilled, enabling biomarker discovery for clinical benefit.
